# Phenotypic Variability of Wool Traits in the Gentile di Puglia Sheep Breed: Implications for Conservation and Sustainable Management

**DOI:** 10.3390/ani16121834

**Published:** 2026-06-14

**Authors:** Maria Gabriela Molina, Grazia Bramante, Claudia Pierini, Medhat S. Saleh, Antonietta D’Onghia, Silverio Grande, Giuseppe Mangini, Silvia Bruno, Virginia Devoto, Elena Ciani, Francesca Maria Sarti, Pasquale De Palo, Vincenzo Landi

**Affiliations:** 1 Facultad de Ciencias Agropecuarias, Universidad Nacional de Córdoba, Córdoba 5000, Argentina; mariagabriela.molina@unc.edu.ar; 2 Department of Veterinary Medicine, University of Bari Aldo Moro, Valenzano, 70010 Bari, Italy; knowout@gmail.com (G.B.); claudia.pierini.620@gmail.com (C.P.); medhat.elshahat@uniba.it (M.S.S.); pasquale.depalo@uniba.it (P.D.P.); 3 Department of Animal Production, Faculty of Agriculture, Benha University, Benha 13736, Egypt; 4 Associazione Regionale Allevatori—Puglia (ARA Puglia), Putignano, 70017 Bari, Italy; a.donghia@ara.puglia.it (A.D.); g.mangini@ara.puglia.it (G.M.); 5 Associazione Nazionale della Pastorizia (ASSONAPA), 00155 Rome, Italy; direzione@assonapa.it; 6 Department of Biosciences, Biotechnologies and Environment, University of Bari Aldo Moro, 70125 Bari, Italy; silviabru3@gmail.com (S.B.); virginiadevotopellegrino@gmail.com (V.D.); elena.ciani@uniba.it (E.C.); 7 Dipartimento di Scienze Agrarie, Alimentari e Ambientali (DSA3), Università degli Studi di Perugia, 35020 Perugia, Italy; francesca.sarti@unipg.it

**Keywords:** Gentile di Puglia, wool quality, fibre diameter, sheep, biodiversity

## Abstract

The Gentile di Puglia sheep breed represents an important component of Southern Italy’s livestock biodiversity, yet its wool traits have not been systematically evaluated for several decades. This study aimed to provide an updated phenotypic assessment of key wool characteristics and to explore the structure of their variability across production contexts. Data were collected from 3089 animals from nine farms over two consecutive years and included fibre diameter, staple length, coefficient of variation in fibre diameter, and clean yield. Descriptive statistics, correlation analysis, principal component analysis, and mixed-effects models were applied to evaluate phenotypic patterns and partition sources of variability. Fibre diameter and staple length showed moderate variability, with most variation occurring within farm-year units, whereas clean yield exhibited a stronger dependence on farm-year conditions. Correlations among traits were generally weak, indicating a high degree of independence. The multivariate analysis revealed a diffuse structure, with no dominant axis explaining most of the variability. Variance component analysis confirmed that production context had a greater influence on clean yield than on fibre-related traits. These results highlight the importance of both conservation and management strategies. The observed variability may provide useful opportunities for future breeding and conservation strategies, while the sensitivity of clean yield to environmental and management factors underscores the need for improved handling practices. Overall, the Gentile di Puglia breed retains a flexible phenotypic profile that supports its conservation and potential valorisation in sustainable production systems.

## Simple Summary

This study describes the wool characteristics of the Gentile di Puglia sheep, a local Italian breed that is currently at risk of extinction. Understanding these traits is important to improve wool quality, support farmers, and preserve biodiversity. Data were collected from several farms over two years, focusing on fibre diameter, fibre uniformity, staple length, and clean wool yield after washing. The results showed that some traits, such as fibre diameter and staple length, mainly vary between individual animals, while clean yield is more influenced by farm conditions and management practices. Relationships among traits were generally weak, meaning that strong phenotypic trade-offs were not evident. This is important for breeding strategies, as it allows farmers to select specific traits without negatively affecting others. Overall, the breed shows a high level of variability, which is a valuable resource for both conservation and production. Improving management practices, especially those affecting wool cleanliness, could significantly increase product value. These findings provide useful information for developing sustainable production systems and promoting the use of local wool in niche markets.

## 1. Introduction

The autochthonous Gentile di Puglia sheep breed represents an important component of the livestock heritage of Southern Italy, with strong historical, cultural, and environmental connections to the region [1]. Historical records describe the presence of fine-wool sheep in this area as early as the first century CE, as documented by classical authors such as Pliny the Elder, Columella, and Varro [2]. According to a number of Roman sources, the Romans employed these animals to enhance Iberian wool production [3]. The breed’s focus switched over time to the production of meat and milk, but its importance for wool production has been restored recently due to interest in local, sustainable materials. Gentile di Puglia continues to be a valuable genetic resource, especially for fine wool production and sustainable agricultural systems in Italy [4]. Following the collapse of the wool industry in the 1960s, population size decreased substantially, and breeding strategies increasingly focused on improving meat production through crossbreeding with other Merino-derived populations [5]. Despite this erosion, genome-wide analyses of Italian sheep diversity have shown that Gentile di Puglia retains a distinct genetic identity within the broader panorama of Italian sheep breeds [6,7].

Despite its historical relevance, the breed has experienced a marked decline over recent decades. According to the most recent census, the Gentile di Puglia population currently consists of approximately 4000 individuals and remains classified as at risk of extinction [8]. The dramatic population decline was driven by multiple factors. First, there was the crisis in the wool industry, caused by a sharp drop in market prices due to the introduction of synthetic fibers. Other reasons included the Gentile di Puglia’s unsuitability for machine milking, determined by udder morphology and low milk yield, as well as the transformation of the production system from transhumance to stationary due to changes in the social structure of the human population [5]. Its unique genetic background and exceptional wool quality, which are marked by ultrafine and fine fiber classes and good fleece homogeneity, have been highlighted in earlier research, underscoring the significance of conservation initiatives to protect this breed from additional genetic erosion [5,9,10,11]. Therefore, the Gentile di Puglia sheep breed should be conserved for the productive, historical, and cultural importance it contributes to the Italian community and global biodiversity.

While wool traits have traditionally represented a key production objective in sheep systems [12], their economic importance has declined in many regions, leading to reduced phenotypic recording and selection pressure. However, renewed interest in natural fibres, product traceability, and sustainable production systems has highlighted the need to re-evaluate local breeds and their productive potential under current conditions.

In this context, the Gentile di Puglia breed represents a particularly relevant case. The last large-scale, population-wide characterization of wool traits in this population dates to the 1950s [13], with a more focused evaluation of fibre quality as a marker of genetic integrity published two decades ago [5]. However, an updated, objective phenotypic assessment of the breed under contemporary management and environmental conditions is currently lacking.

The characterization of phenotypic variation in key traits is essential for supporting conservation strategies and guiding management decisions in endangered populations [14]. In particular, understanding the current distribution and variability of wool traits may help identify realistic opportunities for breed valorization, as well as potential constraints related to fibre quality or production performance.

Therefore, the aim of the present study was to provide an updated phenotypic assessment of key wool traits in the Gentile di Puglia breed, including fibre diameter, staple length, fibre diameter variability, and clean yield. The analysis was conducted on animals from multiple farms located in Southern Italy, representing a range of production systems. This study not only establishes a baseline reference for the breed under current conditions but also provides useful information for future breeding programs focused on wool quality improvement, conservation of genetic diversity, and sustainable management strategies. Furthermore, the results may support conservation initiatives aimed at protecting this important local genetic resource and promoting its use within low-input, biodiversity-oriented production systems.

## 2. Materials and Methods

### 2.1. Dataset and Study Design

Data were collected during 2023 and 2024 from nine commercial sheep farms located in the Apulia (Puglia) region of Southern Italy. The original dataset comprised 3089 individual records. For each animal, sex and age were recorded; age was subsequently classified into two categories: young (<1 year) and adult (≥1 year). Of these, 2908 corresponded to adult females and 177 to adult males. Age distribution of the sampled Gentile di Puglia sheep was assessed using available individual age records. After removing missing records and biologically implausible negative values, age information was available for 1874 animals. Animals younger than 1 year may still exhibit developing fleece characteristics and were also strongly under-represented in the dataset (1.3% of records) and were therefore excluded. Within the age-complete adult subset (*n* = 1818; age range 1.13–16.29 years; mean 6.26 ± 3.81 years; median 5.54 years). The dataset included animals traditionally raised on farms characterized by hot dry summers, mild winters, and extensive or semi-extensive grazing systems. These conditions may contribute to the phenotypic expression of wool traits through differences in pasture availability, seasonal management, and fleece contamination.

To ensure consistency and comparability of phenotypic measurements, subsequent analyses focused on adult animals. Farms were coded as FARM_1, FARM_2, etc., throughout the analyses.

### 2.2. Wool Sampling

Sampling was carried out during the regular annual shearing season, between April and May. In the farms investigated, shearing is routinely performed once per year; therefore, the collected samples corresponded approximately to one annual wool growth period. Sampling was performed at the mid-side region of each animal, approximately at the centre of the flank below the dorsal line. Samples consisted of small wool tufts manually extracted from the fleece, ensuring minimal disturbance of staple structure. Each sample was individually stored in labelled polyethylene bags and transported to the wool laboratory at the Department of Veterinary Science, University of Bari. Sampling was carried out under field conditions and reflects practical on-farm procedures rather than controlled experimental protocols.

### 2.3. Wool Traits

For each animal, the following wool traits were measured:

- fibre diameter (FD, µm)
- staple length (SL, cm)
- coefficient of variation in fibre diameter (CV, %)
- clean yield after washing (CY, %)

Fibre diameter was calculated as the mean of multiple technical measurements per sample, while the coefficient of variation (CV) was derived from the corresponding standard deviation.

### 2.4. Laboratory Analysis

#### 2.4.1. Clean Yield (CY)

Clean yield was determined from subsamples of approximately 10–15 g of greasy wool. Samples were processed following standard washing procedures based on international protocols [15,16,17]. After washing and drying to constant weight, clean yield was calculated as (1)

CY = (fw × 1.16/iw) × 100(1)

where iw is the initial greasy weight and fw is the final dry weight. A regain factor of 16% was applied.

Given the field conditions of sampling and laboratory processing, extreme or implausible values of CY were identified and excluded from the analysis. Only observations within a biologically plausible range (30–90%) were retained for subsequent analyses. The number of observations excluded based on this criterion represented less than 9.1% of the dataset.

#### 2.4.2. Fibre Diameter (FD)

Mean fibre diameter was determined using an opto-electronic fibre diameter meter (FibreLux Micron Meter, FibreLux/Nekan Trading Pty Ltd., Johannesburg, South Africa), based on light diffraction. For each animal, five technical subsamples were measured and averaged to obtain one animal-level estimate.

#### 2.4.3. Staple Length (SL)

Staple length was measured manually using a graduated scale ruler on a randomly selected staple per sample and expressed in centimetres.

### 2.5. Data Processing and Statistical Analysis

Data processing and statistical analyses were performed using R software [18]. To quantify the variability associated with production context, a linear mixed-effects model was fitted for each trait (FD, SL, and CY), including the farm-year combination as a random effect. For CV, the same model specification was fitted on the log-transformed scale due to its skewed distribution and the presence of near-zero values. The model was specified as

y_ij_ = μ + u_i_ + e_ij_ (2)

where y_ij_ is the observation on the j-th animal within the i-th farm-year level, μ is the overall mean, u_i_ is the random effect of the i-th farm-year, assumed u_i_ ~ *N*(0, σ^2^_fy_), and e_ij_ is the residual error, assumed e_ij_ ~ *N*(0, σ^2^_e_). The two farm-year variances were assumed to be independent. Models were fitted by restricted maximum likelihood (REML) using the lme4 package [19]. The proportion of phenotypic variance attributable to the farm-year level, reported throughout the Results as “% farm_year”, was computed as 100 × σ^2^_fy_/(σ^2^_fy_ + σ^2^_e_), corresponding to the intraclass correlation coefficient under the assumed model. Model adequacy was evaluated through simulation-based residual diagnostics (*DHARMa* package [20]), testing residual uniformity, dispersion, and outlier presence. For CV, a generalised linear mixed model with Gamma distribution was also evaluated as an alternative but did not improve the fit and was therefore discarded. This approach allowed the estimation of variance components attributable to differences among farm-year groups while accounting for the hierarchical and unbalanced structure of the dataset.

The empirical distribution of each wool trait was preliminarily evaluated through histograms, density plots, and Shapiro–Wilk tests, complemented by skewness and kurtosis indices. FD (skewness = −0.34) and CY (skewness ≈ 0.00) approached symmetry, whereas SL (skewness = 0.83) and especially CV (skewness = 2.02) were right-skewed; CV included near-zero values and was therefore analysed on the log-transformed scale. Three alternative model specifications were compared for each trait by akaike information criterion (AIC) and Bayesian information criterion (BIC): (i) a linear mixed model on the original scale with a Gaussian link, (ii) a linear mixed model on the log-transformed scale, and (iii) a generalised linear mixed model with Gamma family and log link. For all traits the log-Gaussian specification provided the best fit by AIC and BIC; nevertheless, for FD, SL and CY the corresponding intraclass correlation coefficient (ICC) values on the log-Gaussian and Gaussian specifications differed by less than one percentage point, and the Gaussian model on the original scale was retained for FD, SL and CY to preserve interpretability of variance components in the original units, while the log-Gaussian model was retained for CV. Age of the animal was included as a continuous fixed effect in the linear predictor whenever individual date of birth was available. The decision to include only one random effect (farm-year) was deliberate: with eight farm-year combinations spanning two consecutive shearing seasons, the joint farm-year identifier already captures the main source of contextual clustering, and partitioning farm and year as separate, non-nested random effects led to convergence problems and produced a year-level variance estimated at zero. Pairwise correlations among traits were also re-evaluated through partial correlations adjusted for the farm-year random effect, returning estimates numerically very close to the raw Pearson coefficients (e.g., FD–CV r = −0.258 raw vs. −0.284 adjusted), confirming the robustness of the correlation pattern.

Representativeness of the dataset was evaluated against the official census figures of Gentile di Puglia in Italy. According to the most recent ASSONAPA herdbook data, the breed currently comprises approximately 4000–4500 registered animals distributed across roughly 30–40 active flocks in the Apulia and Basilicata regions. The farms sampled in this study were selected within the area of largest demographic concentration of the breed and represented approximately 25–30% of the active flocks and roughly 70% of the registered female adult population available for shearing in 2023–2024. Although a fully random sampling design was not feasible due to the limited number of active flocks and to operational constraints during the shearing season, the sampling scheme was designed to cover the heterogeneity of farm sizes and management systems characteristic of the breed’s production area. The farm-year level was therefore considered an appropriate stratification factor to capture the joint contribution of geographical, managerial and climatic variability without requiring fine-grained, on-farm environmental recording, which was not available in a standardised form across farms. The number of animals sampled per farm-year unit varied between 21 and 820 records, with a median of 275. To assess the potential influence of this imbalance on variance component estimation, the linear mixed-effects models were also refitted on a subset constructed by randomly down-sampling each farm-year to the median number of records (*n* = 275 per group; total *n* = 1796), and on a subset restricted to farm-year units with at least 100 records (7 of 8 units retained; total *n* = 2825). Under the down-sampling scheme the ICC estimates were 13.6% for FD, 11.6% for SL and 35.2% for CY; under the *n* ≥ 100 restriction they were 10.9% for FD, 13.1% for SL and 35.1% for CY. The variance partitioning was therefore essentially stable across all alternative subsets, with all ICC estimates remaining within a few percentage points of the full-dataset values, indicating that the contribution of farm-year imbalance to the estimated intraclass correlations was modest and that the overall variance partitioning is robust to the unbalanced structure of the dataset.

To verify that the observed multivariate structure was not driven by between-farm-year differences, the principal component analysis was also re-run on within-farm-year scaled residuals (i.e., centring each trait by its farm-year mean). This residual PCA returned variance proportions and loading patterns numerically very close to those obtained on the raw standardised data (PC1 = 32.6% and PC2 = 27.1% on residuals, vs. PC1 = 32.2% and PC2 = 27.3% on raw data), with unchanged sign and ordering of the loadings of FD, CV, SL and CY on PC1 and PC2. This confirms that the diffuse multivariate structure reported throughout the manuscript is an intrinsic feature of the wool phenotype within the breed and is not generated by farm-year clustering.

Given the observational nature of the study, the analytical focus was placed on describing current phenotypic variability and partitioning its main sources rather than on testing complex factorial structures. Pearson correlation coefficients were calculated to evaluate relationships among wool traits. A principal component analysis (PCA) was performed on standardized variables (FD, CV, SL, and CY) to explore the multivariate structure of phenotypic variation.

### 2.6. Ethical Statement

All procedures were conducted during routine farm management practices, and no experimental interventions were applied to animals. Therefore, ethical approval was not required.

## 3. Results

### 3.1. Descriptive Statistics in Males

Descriptive statistics for wool traits in adult male animals (*n* = 177) are reported in Table 1. Across farm-year groups, FD ranged approximately from 18.5 to 24.6 µm, with moderate variability among farms. SL showed noticeable variation, with mean values ranging from 4.7 to 7.1 cm. The CV exhibited high dispersion, with mean values ranging from 3.2% to 33.9%, indicating substantial heterogeneity in fibre uniformity across groups. CY also varied considerably, with mean values between 54.2% and 76.3%. Given the limited number of male records and their uneven distribution across farms and years, these results are presented for descriptive purposes only and were not included in subsequent inferential analyses.

### 3.2. Phenotypic Variation in Females

Descriptive statistics for wool traits in female animals are presented in Table 2. After data filtering, a total of 2846 adult female records were retained for analysis. The CV ranged from 5.4% to 11.2%, indicating differences in fibre uniformity across farms. CY, after exclusion of implausible values, displayed a wide range of values, with mean values between 51.1% and 73.9%, reflecting both biological variation and differences in sampling and processing conditions.

A summary of variance components across traits is presented in Table 3, highlighting the relative contribution of farm-year and residual variability. FD showed a global mean of 23.08 µm. The estimated standard deviation associated with the random effect farm_year was 1.13 µm, whereas the residual standard deviation was 2.84 µm. SL showed a global mean of 5.94 cm. The estimated standard deviation for the random effect farm_year was 0.55 cm, whereas the residual standard deviation was 1.49 cm. As observed for FD, most of the variability was concentrated within sampling units. The proportion of variance attributable to farm_year was approximately 11.81%, indicating moderate structuring. CY analysed on the filtered dataset showed a global mean of 58.93%. The estimated standard deviation for the random effect farm_year was 6.92 percentage points, whereas the residual standard deviation was 9.93 percentage points. In this case, the proportion of variance attributable to farm_year reached approximately 32.69%, indicating a stronger influence of production context compared to FD and SL.

For the CV, residual diagnostics of the log-transformed model indicated reduced influence of extreme observations and improved dispersion behaviour, although residual uniformity remained suboptimal. Therefore, CV was interpreted cautiously and considered as an exploratory indicator of fibre variability. Overall, FD and SL showed similar patterns characterised by moderate between-unit variation and substantial within-unit heterogeneity, whereas CY exhibited a more pronounced structuring across farm-year units. These results indicate that fibre structural traits were less influenced by production context than clean yield, which appeared more sensitive to differences among farm-year conditions.

### 3.3. Relationships Among Wool Traits

Pearson correlation coefficients among wool traits are presented in Table 4. FD showed a weak-to-moderate negative correlation with the CV (r = −0.262, *p* < 0.001), indicating that finer fibres tended to be associated with greater variability in FD. The remaining pairwise associations were statistically significant but weak in magnitude. FD showed a very weak negative correlation with SL (r = −0.058, *p* = 0.0027) and a very weak positive correlation with CY (r = 0.102, *p* < 0.001). Likewise, CV was weakly and positively associated with both SL (r = 0.051, *p* = 0.0084) and CY (r = 0.052, *p* = 0.0068), while the correlation between SL and CY was also weak and positive (r = 0.054, *p* = 0.0048). Overall, although several correlations reached statistical significance, their low magnitude indicates limited biological association among wool traits, suggesting that these variables capture largely distinct aspects of phenotypic variation in the analysed population.

### 3.4. Multivariate Structure of Wool Traits

**Figure 1:**
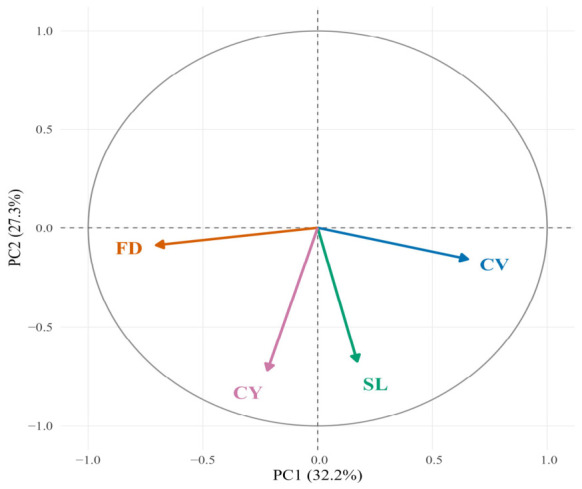
Principal component analysis (PCA) of wool traits based on standardized variables. The arrows represent the fibre diameter (FD), coefficient of variation in fibre diameter (CV), staple length (SL) and clean yield (CY) variables.

PCA was performed on standardized wool traits (FD, CV, SL, and CY) using 2846 adult female records. (Figure 1). The first two principal components explained 59.5% of the total variance, with PC1 accounting for 32.2% and PC2 for 27.3%. In contrast, PC2 was primarily associated with clean yield (CY; loading = 0.77) and staple length (SL; loading = 0.61), indicating that these variables contributed most strongly to this axis and defined a second, largely independent dimension of variation. The graphical representation of variable loadings (Figure 1) showed relatively short vectors and wide angles between variables, consistent with the low pairwise correlations observed. This indicates that the analysed traits contribute complementary and largely independent information to the overall phenotypic structure. Overall, the PCA revealed a diffuse multivariate pattern, with no dominant axis capturing a large proportion of variability, confirming that phenotypic variation in wool traits is distributed across multiple independent dimensions.

The first two components explained 58.9% of the total variance (PC1: 32.2%; PC2: 27.3%). FD and CV mainly contributed to PC1, while CY and SL contributed to PC2. The weak associations among variables, reflected in wide vector angles, suggest that different traits capture distinct aspects of wool characteristics, potentially influenced by both intrinsic fibre properties and external production conditions.

## 4. Discussion

Overall, the results indicate that wool traits in the Gentile di Puglia breed are characterised by moderate levels of structuring across farm-year units and substantial within-group variability. Fibre diameter and staple length showed relatively stable patterns, with most of their variability occurring within farm-year units, whereas clean yield exhibited a stronger dependence on farm-year conditions. This suggests that, while fibre-related traits appeared less dependent on farm-year conditions, clean yield may be more sensitive to contextual factors, including management practices, environmental conditions, and fleece contamination [20]. The mean FD measured in our study (23.08 µm) falls within the average range of FD reported for other breeds. Plowman et et al. [21] reported that the mean of FD was 43.8, 24.4 and 27.2 μm in Churro, Bordaleiro and Merino sheep breeds, respectively in Portugal. The mean FD measured in our study (23.28 µm) falls within the average range of FD reported for other breeds (from 15.80 µm [22] to 25.76 µm [23]. The FD is very low for breeds selected for their wool, as 15.80 µm in Uruguayan Ultrafine Merino sheep [22], 16.86 µm in Australian Merino sheep [24] or 16.9 µm in New Zealand fine wool Merinos [25]. FD measured on a population of Lacaune-Sarda backcross ewes at 6 months of age was 29.9 µm [26]. The mean of SL was 8.5 cm in the Uruguayan Ultrafine Merino sheep [22]. The average of FD, SL and CY were 19.50, 46.0 and 54.66% in the Merino wool in South Africa [27]. The weak correlations among traits and the diffuse multivariate structure revealed by the PCA further support the idea that wool characteristics are not governed by a single dominant gradient but instead represent multiple, largely independent dimensions of variation. This pattern is consistent with previous studies reporting low to moderate phenotypic correlations among wool traits, while genetic correlations reported in the literature may vary across breeds and production systems [28,29,30,31].

The weak-to-moderate negative association between fibre diameter and its coefficient of variation (r = −0.262) is in good agreement with the meta-analytic estimates compiled by Safari et al. [29], which consistently report negative phenotypic and genetic correlations between mean fibre diameter and fibre diameter variability across a wide range of sheep breeds and production contexts. This relationship is commonly interpreted as a biological consequence of follicle activity and primary-to-secondary follicle ratios, whereby finer-wool animals tend to exhibit less uniform fibre populations within the fleece [12,30]. The magnitude observed in the present study is intermediate between the low estimates reported for commercial Merino flocks under intensive recording schemes [29,30] and the stronger correlations occasionally described in less specialized, dual-purpose populations. This is coherent with the intermediate selection history of Gentile di Puglia, which has undergone successive phases of Merino-directed refinement followed by decades of reduced selection pressure on fibre uniformity after the collapse of the wool market [5].

The very weak negative correlation between fibre diameter and staple length (r = −0.058), although statistically significant, is biologically negligible and aligns with the low positive to near-zero phenotypic correlations summarized by Safari et al. [29] for fine and medium wool populations. In selection theory this near-orthogonality between fibre diameter and staple length is particularly favourable, because it implies that length can be increased without inducing a substantial correlated response in fibre thickness, a pattern previously highlighted in Merino-derived systems [29,31]. Similarly, the very small positive correlation observed between fibre diameter and clean yield (r = 0.102) is consistent with the general pattern described in the reviews of Holman & Malau-Aduli [12] and Safari et al. [29], where clean yield is driven predominantly by external contamination (grease, suint, vegetable matter, dirt) rather than by intrinsic fibre geometry. The low magnitude of this correlation further supports the hypothesis that, in our population, clean yield appeared more sensitive to contextual farm-year conditions rather than a fibre-intrinsic one [31]. Our phenotypic negative correlations are consistent with the results of several studies such as (−0.05 ± 0.01, [32]; −0.10 ± 0.02, [33]; −0.16 ± 0.03, [34]; −0.13 ± 0.04, [35]; a weighted mean of −0.05 ± 0.03, [25]. There was a weak positive correlation between FD and SL (0.05) in Alpine Merino sheep [32]. The phenotypic correlations between FD and FDCV were moderate and positive (0.41) in the Lacaune dairy sheep breed [36]. A moderate phenotypic correlation between FD and SL was 0.18 in the Uruguayan Ultrafine Merino sheep [22].

From a practical perspective, these results highlight both opportunities and challenges for the conservation and improvement of the breed. The presence of substantial within-population variability suggests that there may be potential for selection, particularly for fibre diameter and staple length. However, the absence of strong phenotypic associations among traits indicates that major correlated phenotypic responses are not evident in the present dataset [30,37]. At the same time, the sensitivity of clean yield to farm-year conditions underlines the importance of improving management practices and standardising handling and processing procedures to enhance wool quality and market value [29].

Although the present study focuses on phenotypic variability, future research integrating pedigree or genomic information would be valuable to disentangle genetic and environmental sources of variation and to support more targeted breeding strategies.

In the context of local breed conservation, maintaining this phenotypic diversity is of particular importance. Breeding and management strategies should therefore aim to combine moderate, well-controlled selection for wool quality with the preservation of phenotypic and genetic variability, avoiding excessive homogenisation while identifying realistic and context-specific improvement targets. This approach is consistent with current perspectives on the sustainable use of local genetic resources and the role of biodiversity in resilient production systems [14,38]. From a population management perspective, the significant variation within the population, documented here, represents a strategic advantage, not a disadvantage. This indicates that the observed phenotypic variability suggests that an important component of the breed’s adaptive and productive diversity has been maintained despite decades of demographic decline and reduced selection pressure on wool. Breeding programmes coordinated by ASSONAPA and ARA Puglia could therefore benefit from leveraging this variability through low-intensity, multi-trait selection schemes that prioritise the maintenance of biodiversity over rapid homogenization. On the other hand, the strong farm-year dependence of clean yield suggests that improvements in wool quality may be achieved more rapidly through targeted interventions in herd management, nutrition, shearing and post-shearing handling than through genetic selection alone. In this context, recent economic studies have demonstrated that the establishment of innovative wool supply chains based on Gentile di Puglia, integrating traditional weaving techniques and locally branded garments, can substantially close the gross-margin gap between this autochthonous breed and standardised intensively farmed breeds, thus providing a concrete economic incentive for in situ conservation [39].

A structural feature of the present study is the absence of pedigree information consistent and complete enough to fit pedigree-based mixed models. This is a direct consequence of the management framework of the breed: Gentile di Puglia is currently registered under the National Conservation Register coordinated by ASSONAPA, but it is not under any official functional control programme for production traits. Genealogical recording is therefore performed at the level required for conservation status monitoring (census, herdbook entries) and does not include the structured, multi-generational parent-offspring linkage required for the estimation of genetic parameters at the individual-animal level. Within the analytical scope of this study, this implies that the residual variance component captures both additive genetic and permanent environmental variability, which cannot be further partitioned. The development of a structured pedigree recording system remains a priority for the long-term conservation of the breed. This allows for monitoring inbreeding trends, effective population size, unequal use of breeding males, and founder contributions. In parallel, genomic information from SNP genotyping could complement pedigree records by providing genomic relationships and supporting future estimation of genetic parameters and breeding values. A genotyping campaign is currently being planned on a representative subsample of the population within our consortium.

A limitation of the present study is that detailed information on nutrition, seasonal management and climatic conditions was not available in a standardized form across farms. Although the farm-year random effect accounted for overall contextual variability, the individual contribution of specific environmental and management factors could not be formally tested.

## 5. Conclusions

This study provides the first updated phenotypic characterization of wool traits in the Gentile di Puglia sheep breed after more than six decades and confirms the presence of substantial variability under current production conditions. Fibre structural traits were mainly influenced by within farm-year variability, whereas clean yield appeared more sensitive to farm-year context, highlighting the importance of management and handling practices for wool quality improvement. From a conservation perspective, the observed variability represents a valuable resource for maintaining the adaptive and productive potential of the breed. Future strategies should combine moderate and well-controlled breeding approaches with the preservation of phenotypic and genetic diversity, avoiding excessive homogenisation while supporting sustainable production systems adapted to Southern Italy. In parallel, the development of locally valorised wool products, continued phenotypic recording, improved management practices, and the progressive implementation of pedigree and genomic tools may contribute to strengthening long-term in situ conservation programmes and enhancing the economic sustainability of the breed.

## Figures and Tables

**Table 1:** Descriptive statistics of wool traits in adults male animals by farm and year.

Trait ^1^	Farm	Year	N ^2^	Mean ± SD ^3^	Range
CV	FARM_2	2023	8	20.2 ± 18.7	1.8–48.0
CV	FARM_2	2024	8	15.6 ± 15.4	2.6–35.9
CV	FARM_3	2023	37	8.6 ± 11.2	0.4–35.4
CV	FARM_3	2024	11.81	17.3 ± 14.0	1.0–37.6
CV	FARM_4	2023	24	3.2 ± 1.9	1.1–7.4
CV	FARM_4	2024	18	6.2 ± 6.8	1.3–24.3
CV	FARM_5	2023	6	6.2 ± 4.1	2.1–11.9
CV	FARM_5	2024	19	7.5 ± 8.6	1.6–24.8
CV	FARM_6	2024	6	14.4 ± 13.6	3.1–35.8
CV	FARM_7	2023	13	9.3 ± 9.8	1.1–33.9
CV	FARM_7	2024	19	5.5 ± 7.1	0.4–34.0
CV	FARM_8	2024	5	8.4 ± 8.4	2.9–23.2
CV	FARM_9	2024	2	33.9 ± 2.9	31.9–35.9
CY	FARM_2	2023	8	70.0 ± 6.6	62.2–79.0
CY	FARM_2	2024	7	62.0 ± 8.1	49.2–70.6
CY	FARM_3	2023	35	64.5 ± 14.5	35.8–87.9
CY	FARM_3	2024	10	54.2 ± 3.8	49.9–61.6
CY	FARM_4	2023	23	75.3 ± 9.1	53.4–87.7
CY	FARM_4	2024	18	62.7 ± 12.1	46.9–88.9
CY	FARM_5	2023	5	65.0 ± 6.1	54.9–71.2
CY	FARM_5	2024	19	57.8 ± 6.0	49.1–71.0
CY	FARM_6	2024	6	64.4 ± 19.1	38.1–89.5
CY	FARM_7	2023	13	68.4 ± 8.3	45.5–75.9
CY	FARM_7	2024	18	59.2 ± 8.7	41.8–72.5
CY	FARM_8	2024	5	76.3 ± 10.0	63.2–87.7
FD	FARM_2	2023	8	18.5 ± 3.7	12.8–23.5
FD	FARM_2	2024	8	19.6 ± 2.6	16.2–22.5
FD	FARM_3	2023	37	23.5 ± 4.3	13.0–32.3
FD	FARM_3	2024	12	22.7 ± 3.3	17.9–27.3
FD	FARM_4	2023	24	23.8 ± 2.2	18.0–28.8
FD	FARM_4	2024	18	22.9 ± 2.1	20.3–29.2
FD	FARM_5	2023	6	24.6 ± 3.4	20.7–28.8
FD	FARM_5	2024	19	22.1 ± 2.5	18.0–26.5
FD	FARM_6	2024	6	20.3 ± 4.4	13.7–26.3
FD	FARM_7	2023	13	23.2 ± 2.9	16.7–26.7
FD	FARM_7	2024	19	21.5 ± 2.9	16.6–26.6
FD	FARM_8	2024	5	22.3 ± 1.4	20.3–23.8
FD	FARM_9	2024	2	18.8 ± 0.3	18.6–19.0
SL	FARM_2	2023	8	6.1 ± 1.6	4.3–9.0
SL	FARM_2	2024	8	5.5 ± 2.0	2.0–8.2
SL	FARM_3	2023	37	7.1 ± 2.8	2.0–13.0
SL	FARM_3	2024	12	5.0 ± 1.1	2.8–6.5
SL	FARM_4	2023	24	5.6 ± 1.6	2.0–8.5
SL	FARM_4	2024	18	4.7 ± 1.3	2.7–6.7
SL	FARM_5	2023	6	5.9 ± 1.8	3.9–8.7
SL	FARM_5	2024	19	4.9 ± 1.1	3.3–6.7
SL	FARM_6	2024	6	5.6 ± 2.2	3.3–8.4
SL	FARM_7	2023	13	6.5 ± 1.6	3.5–9.1
SL	FARM_7	2024	19	4.8 ± 1.3	2.8–7.9
SL	FARM_8	2024	5	7.0 ± 2.3	3.9–9.2
SL	FARM_9	2024	2	3.2 ± 0.1	3.1–3.3

^1^ FD: fibre diameter (µm); CV: coefficient of variation in fibre diameter (%); SL: staple length (cm); CY: clean yield (%). ^2^ N: number of observations. ^3^ SD: standard deviation.

**Table 2:** Descriptive statistics of wool traits in adult female animals by farm and year.

Trait ^1^	Farm	Year	N ^2^	Mean ± SD ^3^	Range
CV	FARM_1	2024	22	9.3 ± 8.8	2.1–35.7
CV	FARM_2	2023	287	10.6 ± 10.8	0.9–40.4
CV	FARM_3	2023	852	5.4 ± 6.6	0.0–37.3
CV	FARM_4	2023	467	6.3 ± 5.2	0.5–37.8
CV	FARM_5	2023	594	7.8 ± 9.5	0.2–52.6
CV	FARM_6	2024	271	11.2 ± 9.9	0.6–40.9
CV	FARM_7	2023	208	7.2 ± 6.4	0.5–31.8
CV	FARM_8	2024	207	10.3 ± 10.2	0.6–38.7
CY	FARM_1	2024	21	70.1 ± 5.7	60.9–82.2
CY	FARM_2	2023	275	71.3 ± 8.6	43.1–89.8
CY	FARM_3	2023	694	73.9 ± 14.8	30.0–89.9
CY	FARM_4	2023	450	63.3 ± 11.6	35.6–89.8
CY	FARM_5	2023	586	64.3 ± 9.1	34.8–89.0
CY	FARM_6	2024	265	69.0 ± 9.6	41.7–89.7
CY	FARM_7	2023	204	51.1 ± 9.2	30.4–80.0
CY	FARM_8	2024	206	58.9 ± 9.3	38.2–84.3
FD	FARM_1	2024	22	25.2 ± 2.5	21.3–29.9
FD	FARM_2	2023	287	24.4 ± 3.2	13.7–33.3
FD	FARM_3	2023	852	23.3 ± 2.9	12.4–33.4
FD	FARM_4	2023	467	22.7 ± 2.0	14.2–29.5
FD	FARM_5	2023	594	22.6 ± 3.2	11.0–33.3
FD	FARM_6	2024	271	23.8 ± 3.2	13.4–30.9
FD	FARM_7	2023	208	23.5 ± 2.2	16.4–31.3
FD	FARM_8	2024	207	21.2 ± 2.9	11.9–27.8
SL	FARM_1	2024	22	5.8 ± 1.3	3.4–9.5
SL	FARM_2	2023	287	6.6 ± 1.5	3.7–14.3
SL	FARM_3	2023	852	6.1 ± 1.3	2.0–13.4
SL	FARM_4	2023	467	5.3 ± 1.3	2.0–11.2
SL	FARM_5	2023	594	6.1 ± 1.7	2.1–16.5
SL	FARM_6	2024	271	5.0 ± 1.7	2.0–13.2
SL	FARM_7	2023	208	6.1 ± 1.2	3.2–9.8
SL	FARM_8	2024	207	6.5 ± 1.9	1.5–12.9

^1^ FD: fibre diameter (µm); CV: coefficient of variation of fibre diameter (%); SL: staple length (cm); CY: clean yield (%). ^2^ N: number of observations. ^3^ SD: standard deviation.

**Table 3:** Variance components of wool traits based on mixed-effects models.

Trait ^1^	Mean	SD (Farm_Year) ^2^	SD (Residual)	% Farm_Year
FD	23.08	1.13	2.84	13.57
SL	5.94	0.55	1.49	12.0
CY	58.93	6.92	9.93	32.69

^1^ FD: fibre diameter (µm); SL: staple length (cm); CY: clean yield (%). ^2^ SD: standard deviation. Variance components were estimated using mixed-effects models including farm-year as a random effect.

**Table 4:** Pearson correlation coefficients (r) and corresponding *p*-values among wool traits in female animals.

Trait Pair ^1^	r ^2^	*p*-Value
FD–CV	−0.262	<0.001
FD–SL	−0.058	0.0027
FD–CY	0.102	<0.001
CV–SL	0.051	0.0084
SL–CY	0.054	0.0048
CV–CY	0.052	0.0068

^1^ FD: fibre diameter; CV: coefficient of variation in fibre diameter; SL: staple length; CY: clean yield. ^2^ Correlation coefficients (r) are shown together with their corresponding *p*-values.

## Data Availability

The data presented in this study are available from the corresponding author upon reasonable request.

## Abbreviations

The following abbreviations are used in this manuscript:

FD	Fibre diameter
CV	Coefficient of variation in fibre diameter
SL	Staple length
CY	Clean yield
PCA	Principal component analysis
